# Supra-Annular Versus Intra-Annular Self-Expanding Valves in Small Aortic Annulus: A Propensity Score-Matched Study

**DOI:** 10.1016/j.shj.2024.100334

**Published:** 2024-07-08

**Authors:** Michel Pompeu Sá, Danial Ahmad, Yisi Wang, Floyd Thoma, Amber Makani, Dustin Kliner, Catalin Toma, David West, Derek Serna-Gallegos, Ibrahim Sultan

**Affiliations:** a Department of Cardiothoracic Surgery, University of Pittsburgh, Pittsburgh, Pennsylvania, USA; b UPMC Heart and Vascular Institute, University of Pittsburgh Medical Center, Pittsburgh, Pennsylvania, USA; c Department of Interventional Cardiology, University of Pittsburgh, Pittsburgh, Pennsylvania, USA

**Keywords:** Aortic valve disease, Aortic valve replacement, Aortic valve stenosis, Cardiac surgical procedures, Cardiovascular surgical procedures, Heart valve diseases, Heart valve prosthesis implantation, Transcatheter aortic valve replacement

## Abstract

**Background:**

Transcatheter aortic valve replacement (TAVR) with self-expanding valves (SEVs) may have different outcomes with supra-annular valves (SAVs) or intra-annular valves (IAVs) in patients with small aortic annuli (SAA), but this topic remains underexplored. We aimed to evaluate outcomes between different SEVs, namely SAVs (CoreValve/Evolut R/PRO/PRO+/FX) vs. IAVs (Portico/Navitor).

**Methods:**

Single-center data with patients with SAA (maximum diameter <23 mm) who underwent TAVR from 2013 to 2023 with SEVs, followed by 1:1 propensity score matching (PSM).

**Results:**

We obtained 86 PSM pairs with median age of 83.0 years (SAVs) and 82.0 years (IAVs), with women representing 77.6% of the PSM cohort. After TAVR, we did not find statistically significant differences for the following outcomes: Valve Academic Research Consortium-3 periprocedural mortality, technical success, device success, clinical efficacy, and rates of paravalvular leak were not statistically significantly different, but we found higher rates of permanent pacemaker implantation in the IAV group (1.2 vs. 8.1%; *p* = 0.029). Despite the larger indexed effective orifice area with SAVs (median 1.0 vs. 0.8 cm^2^/m^2^, *p* = 0.001), we did not find statistically significant differences between the groups in terms of residual mean gradients >20 mmHg (0.0 vs. 2.3%, *p* = 0.155), and severe prosthesis-patient mismatch (2.3 vs. 5.8%, *p* = 0.390). No statistically significant difference was observed in survival (log-rank *p* = 0.950) and stroke (*p* = 0.6547) between patients who received SAVs and IAVs. For patients with SAA, TAVR with SEV devices is safe.

**Conclusions:**

IAVs and SAVs are associated with comparable device performance in terms of hemodynamic structural and nonstructural dysfunction. Randomized data are needed to validate these findings and guide informed device selection.

## Highlights

• Self-expanding valve (SEV) with supra-annular valve (SAV) or intra-annular valve (IAV) design in small aortic annulus remain underexplored.
• In 86 propensity score matching pairs, SAV and IAV did not present differences in Valve Academic Research Consortium-3 outcomes.
• Larger indexed effective orifice area with SAV, but no differences in residual mean gradients >20 mmHg.
• No differences in severe prosthesis-patient mismatch between SEV-SAV and SEV-IAV.
• No statistically significant difference in survival between stroke SAVs and IAVs.

## Introduction

Transcatheter aortic valve replacement (TAVR) has become the most disruptive technology in the management of severe aortic stenosis (AS) over the last 20 years and has become the gold-standard treatment for patients at high, intermediate, and even low surgical risk. Although the CoreValve/Evolut (Medtronic, Dublin, Ireland) and SAPIEN (Edwards Lifesciences, Irvine, California) TAVR platforms are the most used commercial devices with their performance constantly put under the microscope,^1^, ^2^, ^3^, ^4^ new platforms such as Portico/Navitor (Abbott Vascular, Chicago, Illinois) arose on the structural heart market.^5^

The presence of a small aortic annulus (SAA) poses a considerable challenge in the management of patients with severe AS, especially in elderly women, in whom it is a very frequent finding and associated with increased risk of prosthesis-patient mismatch (PPM),^6^ which in turn is associated with an increased risk of overall mortality proportionally to its severity, suboptimal valve hemodynamics, and less left ventricular mass regression after both TAVR^7^ and surgical aortic valve replacement.^8^^,^^9^

The hemodynamic performance of transcatheter heart valves (THVs) has been considered a reliable predictor of long-term clinical outcomes.^7^^,^^10^ Within the SAA subgroup, evidence from observational registries indicates better hemodynamic performance for TAVR, with self-expanding valves (SEVs) outperforming balloon-expandable valves (BEVs).^3^^,^^11^, ^12^, ^13^ Noteworthy, these studies^3^^,^^11^, ^12^, ^13^ compare SEVs with a supra-annular design against BEVs, which have an intra-annular design. Therefore, behind the SEV-vs.-BEV comparison lies a comparison between supra-annular valves (SAVs) and intra-annular valves (IAVs). This is important because IAVs are associated with lower effective orifice area (EOA) and, thus, tend to present higher post-TAVR gradients and higher rates of PPM.^6^ In this scenario, a question remains underexplored: are there significant differences in the performance of SAVs and IAVs if both platforms are SEVs?

In this context, we aimed to compare the hemodynamic and clinical performance of 2 SEV platforms in contemporary TAVR practice: the Medtronic SAV (CoreValve, Evolut R, Evolut PRO/PRO+ and Evolut FX) vs. the Abbott Vascular IAV (Portico/Navitor) in patients with SAA.

## Methods

### Study Design

This study was an observational, retrospective analysis of the institutional Transcatheter Valve Therapies database. Standard American College of Cardiology/Society of Thoracic Surgeons Transcatheter Valve Therapies registry definitions and terminologies were used in this study. All patients with SAA who underwent transfemoral TAVR in a native aortic valve from 2013 to 2023 with SEVs were included in this analysis. Patients who underwent valve-in-valve TAVR (any previous aortic valve intervention), pure aortic regurgitation, patients <18 years of age, and those with no follow-up echocardiographic data were excluded.

### Definitions

SAA was defined as an aortic annulus with a maximum diameter of <23 mm on computed tomographic measurement.^6^ Valve performance, procedural, and clinical outcomes were defined on the basis of the Valve Academic Research Consortium-3 (VARC-3) definitions.^14^ Bioprosthetic valve dysfunction was defined as hemodynamic structural valve dysfunction (HSVD) if the mean pressure gradient was ≥20 mmHg or nonstructural valve dysfunction (NSVD) if severe PPM or moderate/severe paravalvular leak (PVL) was present. Technical success was defined as follows: 1) freedom from mortality; 2) successful access, delivery of the device, and retrieval of the delivery system; 3) correct positioning of a single prosthetic heart valve into the proper anatomical location; and 4) freedom from surgery or intervention related to the device (excluding permanent pacemaker) or a major vascular, access-related, or cardiac structural complication at exit from the procedure room. Device success was defined as follows: 1) technical success; 2) 30-day freedom from mortality; 3) 30-day freedom from surgery or intervention related to the device (excluding permanent pacemaker) or a major vascular, access-related, or cardiac structural complication; and 4) intended performance of the valve (mean gradient <20 mmHg, peak velocity <3 m/s, Doppler velocity index >0.25, and less than moderate aortic regurgitation). Periprocedural mortality was defined as death meeting one of the following criteria: occurring <30 days after the index procedure or >30 days but during the index hospitalization. Clinical efficacy (at 1 year and thereafter) was defined as: freedom from all-cause mortality; freedom from stroke; freedom from hospitalization for procedure- or valve-related causes; freedom from the Kansas City Cardiomyopathy Questionnaire (KCCQ) overall summary score <45 or decline from the baseline of >10 points.

### Outcomes

The primary outcomes were periprocedural mortality, technical success, device success, clinical efficacy as defined by VARC-3 criteria, survival, and stroke rates at 2 years.

The secondary outcomes were the following (at 30 days and 1 year): permanent pacemaker implantation (PPI), moderate/severe PVL, residual mean gradient, dimensionless index, indexed EOA, presence of PPM, and KCCQ-12 score.

### Statistical Analysis

Unmatched and propensity-matched baseline characteristics, clinical characteristics, and echocardiographic outcomes at 30 days as well as 1 year were compared between the cohorts. The nearest neighbor (1:1) propensity score matching (PSM) method was used to match patients on selected baseline characteristics. Continuous data are presented as mean ± standard deviation for normally distributed data or median and interquartile range (IQR) for non-normally distributed data. Categorical data are presented as frequency and percentage. Normally distributed continuous data were analyzed using Student's t test, while non-normally distributed continuous data were analyzed with the Mann-Whitney U test. Categorical data were compared via the chi-square or Fisher's exact test as appropriate. A univariable Cox regression model for mortality as well as a Fine and Grey competing risk model for stroke were created.

All tests were two-sided, with an alpha level of 0.05 set to indicate statistical significance. Unadjusted and propensity-matched survival estimates obtained through Kaplan-Meier analysis were compared using logrank statistics. All statistical analyses were performed using SAS/STAT Version 15.2 (SAS Institute Inc, Cary, NC, USA).

## Results

### Study Population

After applying the predefined exclusion criteria, we identified 583 patients with SAA who underwent TAVR with SEVs using either SAV (n = 477) or IAV (n = 106) between 2013 and 2023. PSM (accounting for demographic, clinical, and anatomical characteristics) resulted in 86 matched pairs of patients receiving either SAV or IAV platform. Baseline patient characteristics of the matched and unmatched cohorts are presented in Table 1. The matched cohort was predominantly female (83.7 and 89.5%) and White (93.0 and 95.3%), with a median age of 83.0 and 82.0 years. The adjusted cohort was well balanced, except that we still observed a statistically significant difference in the median Society of Thoracic Surgeons mortality score, which was higher in the IAV group in comparison with the SAV group (median for SAV 3.2, IQR 2.3-5.3 vs. median for IAV 4.2, IQR 3.0-5.8, *p* = 0.005).

**Table 1:** Baseline characteristics in unmatched and matched populations

Baseline characteristics	Unmatched population			Propensity-matched population		
	Supra-annular (n = 477)	Intra-annular (n = 106)	*P* value	Supra-annular (n = 86)	Intra-annular (n = 86)	*P* value
Age, years	81.0 (76.0-86.0)	82.0 (77.0-89.0)	0.056	83.0 (76.0-87.0)	82.0 (78.0-89.0)	0.675
Sex, female	400 (83.6%)	90 (90.6%)	0.080	72 (83.7%)	77 (89.5%)	0.261
Race			0.081			0.778
White	443 (92.9%)	100 (94.3%)		80 (93.0%)	82 (95.3%)	
Black	11 (2.3%)	5 (4.7%)		4 (4.7%)	3 (3.5%)	
Other	23 (4.8%)	1 (0.9%)		2 (2.3%)	1 (1.2%)	
BMI	28.6 (24.2-33.6)	28.5 (23.5-35.1)	0.953	29.1 (25.5-34.4)	29.2 (23.7-35.5)	0.829
BSA	1.80 ± 0.23	1.79 ± 0.25	0.668	1.82 ± 0.21	1.80 ± 0.25	0.652
NYHA class (2 wk pre-TAVR)			0.734			0.207
I	16 (3.4%)	5 (4.7%)		3 (3.5%)	3 (3.5%)	
II	257 (53.9%)	52 (49.1%)		55 (64.0%)	43 (50.0%)	
III	183 (38.4%)	45 (42.5%)		27 (31.4%)	36 (41.9%)	
IV	21 (4.4%)	4 (3.8%)		1 (1.2%)	4 (4.7%)	
Hypertension	437 (91.6%)	101 (95.3%)	0.200	78 (90.7%)	83 (96.5%)	0.119
Diabetes	147 (30.8%)	30 (28.3%)	0.610	24 (27.9%)	27 (31.4%)	0.616
COPD	119 (24.9%)	32 (30.2%)	0.265	28 (32.6%)	26 (30.2%)	0.742
Current dialysis	10 (2.1%)	6 (5.7%)	0.042	2 (2.3%)	5 (5.8%)	0.247
Preprocedural creatine	1.0 (0.8-1.2)	1.0 (0.8-1.3)	0.246	0.9 (0.8-1.1)	1.0 (0.8-1.3)	0.069
Atrial fibrillation	97 (20.3%)	31 (29.2%)	0.045	26 (30.2%)	28 (32.6%)	0.742
Prior PAD	61 (12.8%)	11 (10.4%)	0.490	11 (12.8%)	8 (9.3%)	0.465
Prior stroke	55 (11.5%)	13 (12.3%)	0.831	11 (12.8%)	10 (11.6%)	0.815
Urgent/emergent TAVR	3 (0.6%)	3 (2.8%)	0.042	1 (1.2%)	2 (2.3%)	0.560
Prior CABG/PCI	146 (30.6%)	42 (39.6%)	0.072	36 (41.9%)	32 (37.2%)	0.532
Unable to walk	21 (4.4%)	11 (10.4%)	0.014	6 (7.0%)	10 (11.6%)	0.293
MR moderate	10 (2.1%)	0 (0.0%)	0.132	0 (0.0%)	0 (0.0%)	N/A
Previous RBBB/LBBB	135 (28.3%)	35 (33.0%)	0.333	22 (25.8)	32 (32.5%)	0.313
LVEF (%)	63.0 (55.0-65.0)	63.0 (58.0-63.0)	0.203	63.0 (58.0-65.0)	63.0 (58.0-63.0)	0.203
STS PROM (median)	3.4 (2.3-5.6)	4.2 (2.9-5.8)	0.002	3.2 (2.3-5.3)	4.2 (3.0-5.8)	0.005
AV area (cm^2^)	0.6 (0.5-0.7)	0.7 (0.6-0.8)	0.026	0.7 (0.6-0.8)	0.7 (0.6-0.8)	0.762
AV mean gradient (mmHg)	43.0 (39.0-52.0)	45.0 (40.0-57.0)	0.099	45.0 (40.0-53.0)	44.5 (40.0-52.0)	0.788
AV annulus diameter (mm)	21.7 (21.0-22.3)	22.0 (21.2-22.5)	0.011	22.3 (21.2-22.7)	22.0 (21.2-22.4)	0.091
AV annulus area (mm^2^)	367.0 (344.0-389.0)	371.0 (344.0-390.0)	0.797	386 (347-399)	370 (341-390)	0.059
AV annulus perimeter (mm)	69.4 (67.0-71.0)	70 (67.2-71.5)	0.4161	70.6 (67.2-71.6)	70.6 (67.4-71.5)	0.352
Bicuspid aortic valve	7 (1.5%)	0 (0.0%)	N/A	1 (1.2%)	0 (0.0%)	0.315
KCCQ-12 overall	58.7 (37.0-80.0)	51.5 (35.0-71.0)	0.079	59.0 (36.0-82.0)	50.5 (34.0-70.5)	0.117

Abbreviations: AV, aortic valve; BMI, body mass index; BSA, body surface area; CABG, coronary artery bypass graft; COPD, chronic obstructive pulmonary disease; KCCQ-12, Kansas City Cardiomyopathy Questionnaire; LBBB, left bundle branch block; LVEF, left ventricular ejection fraction; MR, mitral regurgitation; N/A, nonapplicable; NYHA, New York Heart Association; PAD, peripheral artery disease; PCI, percutaneous coronary intervention; RBBB, right bundle branch block; STS-PROM, Society of Thoracic Surgeons-Predicted Risk of Mortality; TAVR, transcatheter aortic valve replacement.

The median values for annulus diameter (mm), annulus area (mm^2^), and annulus perimeter (mm) in SAV and IAV groups were the following: 22.3 vs. 22.0 (*p* = 0.091), 386 vs. 370 (*p* = 0.059), and 70.6 vs. 70.6 (*p* = 0.352), respectively.

Regarding the THV sizes, the most common SAV implanted was 26 mm (82.8%), followed by 29 mm (11.1%) and 23 mm (6.1%), while the most common IAV implanted was 25 mm (63.2%), followed by 27 mm (20.8%) and 23 mm (16.0%).

### Primary Outcomes

The VARC-3-defined composite outcomes of technical success, device success, and clinical efficacy (Table 2) were comparable between groups (SAV 95.3% vs. IAV 93.0% [*p* = 0.514], SAV 95.3% vs. IAV 93.0% [*p* = 0.514], and SAV 73.3% vs. IAV 70.9% [*p* = 0.733], respectively).

**Table 2:** Primary outcomes

Outcomes	Unmatched population			Propensity-matched population		
	Supra-annular (n = 477)	Intra-annular (n = 106)	*P* value	Supra-annular (n = 86)	Intra-annular (n = 86)	*P* value
VARC-3 Periprocedural mortality	13 (2.7%)	5 (4.7%)	0.283	3 (3.5%)	4 (4.7%)	0.699
VARC-3 Technical success	447 (93.7%)	99 (93.4%)	0.904	82 (95.3%)	80 (93.0%)	0.514
ARC-3 Device success	441 (92.5%)	99 (93.4%)	0.736	82 (95.3%)	80 (93.0%)	0.514
VARC-3 Clinical efficacy	345 (72.3%)	74 (69.8%)	0.602	63 (73.3%)	61 (70.9%)	0.733

Abbreviation: VARC-3, Valve Academic Research Consortium 3.

The VARC-3-defined outcomes of periprocedural mortality, early mortality, and late mortality (Table 2) were comparable between groups (SAV 3.5% vs. IAV 4.7% [*p* = 0.699], SAV 8.1% vs. IAV 5.8% [*p* = 0.549], SAV 2.3% vs. IAV 1.2% [*p* = 0.560], respectively).

**Figure 1:**
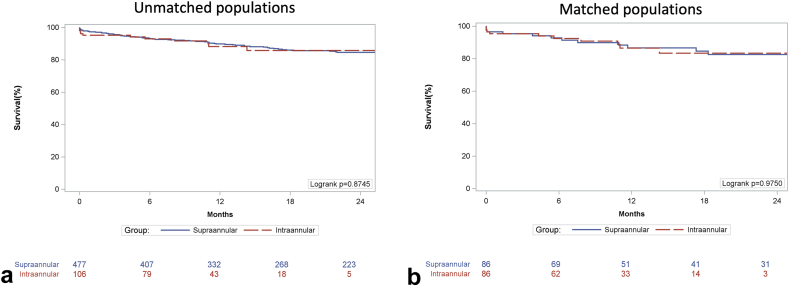
Kaplan-Meier survival analysis in (a) unmatched and (b) matched populations.

**Figure 2:**
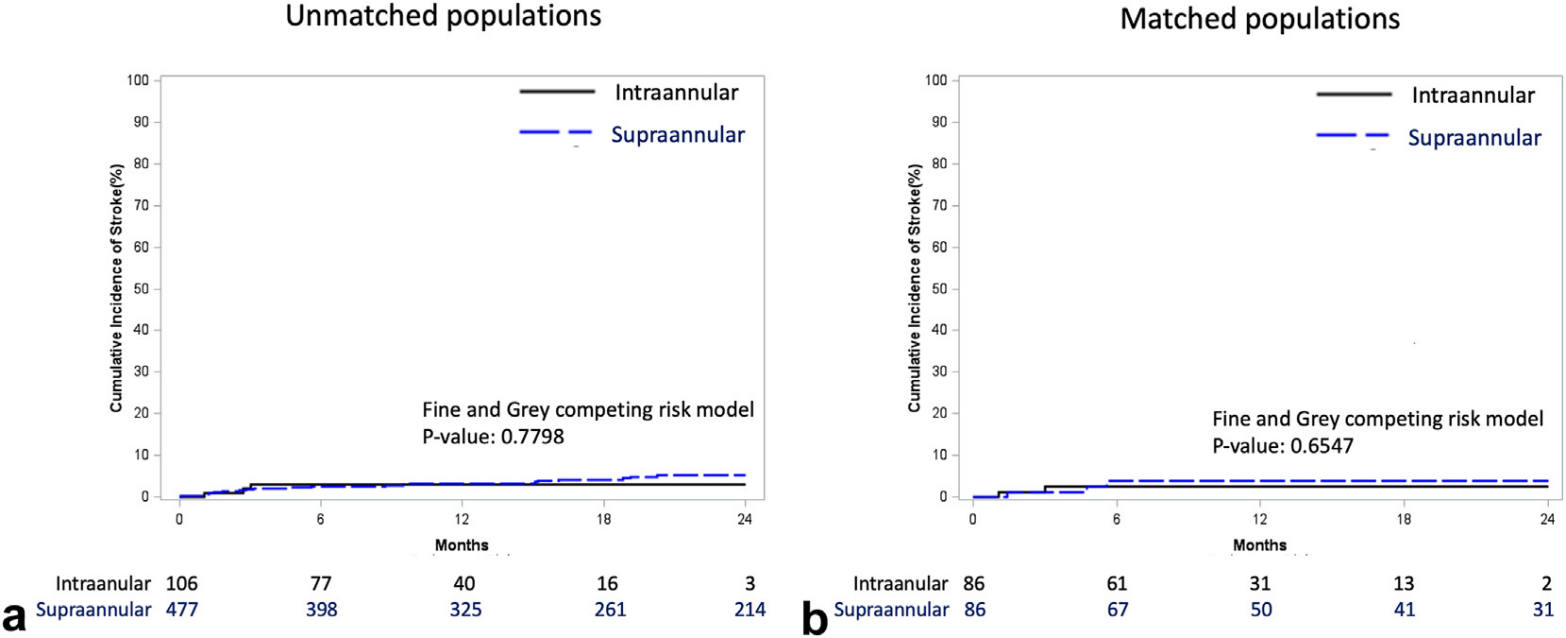
Cumulative incidence of stroke in (a) unmatched and (b) matched populations.

Kaplan-Meier time-to-event analysis at 2 years confirmed comparable rates of all-cause death (Figure 1) for both unmatched (SAV 15.3% vs. IAV 14.2%, P*log-rank* = 0.8745) and matched cohorts (SAV 17.4% vs. IAV 16.6%, P*log-rank* = 0.9750). Furthermore, the cumulative incidence of stroke at 2 years was comparable (Figure 2) for both unmatched (SAV 5.2% vs. IAV 3.0%, *p* = 0.7798) and matched cohorts (SAV 3.8% vs. IAV 2.5%, *p* = 0.6547).

### Secondary Outcomes

Clinical and hemodynamic secondary outcomes are presented in Table 3. No statistically significant difference between the groups was observed for significant PVL at 30 days. However, the 30-day incidence of PPI was statistically significantly higher in the IAV group compared with the SAV group (SAV 1.2% vs. IAV 8.1%, *p* = 0.029).

**Table 3:** Secondary outcomes

Outcomes	Unmatched population			Propensity-matched population		
	Supra-annular	Intra-annular	*P* value	Supra-annular	Intra-annular	*P* value
30-d permanent pacemaker implantation	26 (5.5%)	9 (8.5%)	0.233	1 (1.2%)	7 (8.1%)	0.029
30-d paravalvular leak (moderate/severe)	3 (0.6%)	2 (1.9%)	0.491	0 (0.0%)	1 (1.2%)	0.316
30-d residual mean gradient >20 mmHg	7 (1.5%)	2 (1.9%)	0.751	0 (0.0%)	2 (2.3%)	0.155
30-d residual mean gradient (mmHg)	9.0 (6.0-12.0)	9.0 (7.0-13.0)	0.190	9.0 (6.0-12.0)	9.0 (7.0-12.0)	0.500
1-y residual mean gradient (mmHg)	7.0 (5.0-10.0)	8.0 (7.0-13.0)	0.175	8.0 (6.0-10.0)	8.5 (8.0-13.0)	0.193
30-d dimensionless index	0.6 (0.5-0.7)	0.6 (0.5-0.7)	1.000	0.6 (0.5-0.8)	0.6 (0.5-0.7)	0.343
1-y dimensionless index	0.6 (0.5-0.7)	0.6 (0.5-0.7)	1.000	0.6 (0.5-0.7)	0.6 (0.5-0.7)	1.000
30-d indexed EOA∗ (cm^2^/m^2^; median-IQR)	1.0 (0.8-1.2)	0.9 (0.7-1.1)	0.001	1.0 (0.9-1.2)	0.8 (0.7-1.1)	0.009
30-d any prosthesis-patient mismatch†	63 (12.8%)	26 (24.5%)	0.005	14 (16.3%)	20 (23.2%)	0.390
None	416 (87.2%)	80 (75.5%)		72 (83.7%)	66 (76.7%)	
Moderate	44 (9.2%)	21 (19.8%)		12 (14.0%)	15 (17.4%)	
Severe	17 (3.6%)	5 (4.7%)		2 (2.3%)	5 (5.8%)	
1-y KCCQ-12 score (median-IQR)	89.0 (71.0-98.0)	80.5 (67.0-93.5)	0.021	81.0 (59.0-97.5)	77.0 (67.0-93.0)	0.587

Abbreviations: EOA, effective orifice area; IQR, interquartile range; KCCQ-12, Kansas City Cardiomyopathy Questionnaire.

∗ EOA according to measurements obtained by Doppler echocardiography and indexed to body surface area.

† Based on thresholds considering body surface area and body mass index (presence/absence of obesity).

Although we observed a statistically significant difference in the median values of indexed EOA favoring the SAV group when compared with the IAV group (SAV 1.0 cm^2^/m^2^, IQR 0.9-1.2 vs. IAV 0.8 cm^2^/m^2^, IQR 0.7-1.1, *p* = 0.009), we did not observe statistically significant differences in the median values of residual mean gradients at 30 days (SAV 9.0 mmHg, IQR 6.0-12.0 vs. IAV 9.0 mmHg, IQR 7.0-12.0, *p* = 0.500) and at 1 year (SAV 8.0 mmHg, IQR 6.0-10.0 vs. IAV 8.5 mmHg, IQR 8.0-13.0, *p* = 0.193). At 30 days, no statistically significant difference was observed in the rates of residual mean gradient >20 mmHg (SAV 0.0% vs. IAV 2.3%, *p* = 0.155). Additionally, we did not observe a statistically significant difference in the rates of severe PPM between the groups at 30 days (SAV 2.3% vs. IAV 5.8%, *p* = 0.390) and in the median values of dimensionless index (SAV 0.6 mmHg, IQR 0.5-0.8 vs. IAV 0.6 mmHg, IQR 0.5-0.7, *p* = 0.343). Furthermore, no statistically significant difference between the groups was observed for KCCQ-12 scores at 1 year (SAV 81.0, IQR 59-97.5 vs. IAV 77.0, IQR 67.0-93.0, *p* = 0.587).

## Discussion

### Summary of Findings

This PSM analysis was carried out to investigate the performance of 2 SEV platforms in patients with SAA. The main findings are as follows:

1) TAVR performed with SAV and IAV was found to have no statistically significant differences in VARC-3 periprocedural mortality, technical success, device success, clinical efficacy, 2-year survival, and 2-year stroke;
2) Despite higher indexed EOA in the SAV group, there were no statistically significant differences in 30-day and 1-year residual mean gradients and dimensionless index between SAV and IAV;
3) We did not find a statistically significant difference in the rates of residual mean gradient >20 mmHg, thus no statistically significant difference in terms of HSVD;
3) There were no statistically significant differences in the rates of moderate/severe PVL and severe PPM between SAV and IAV at 30 days, thus no statistically significant difference in terms of NSVD;
4) SAV was associated with lower rates of PPI in comparison with IAV at 30 days;
5) At 1-year, SAV and IAV were associated with comparable functional status and quality of life, as evidenced by the 1-year KCCQ-12 scores for both groups.

### Comments

Comparative follow-up data, including TAVR platforms, in the setting of SAA is scarcely represented in randomized trials. The sole trial comparing 2 TAVR platforms in the context of SAA was the recently published SMART trial,^15^ which randomly assigned a total of 716 patients with symptomatic severe AS and an aortic valve (AV) annulus area of 430 mm^2^ or less (mean age 80 years; 87% women; mean Society of Thoracic Surgeons-Predicted Risk of Mortality score 3.3%) to receive either an Evolut PRO/PRO+/FX (SEV-SAV) or SAPIEN 3/3 Ultra (BEV-IAV). The Kaplan-Meier estimate of the percentage of patients who died, had a disabling stroke, or were rehospitalized for heart failure through 12 months was 9.4% with SEV and 10.6% with BEV (*p* <0.001 for noninferiority). The Kaplan-Meier estimate of the percentage of patients with bioprosthetic valve dysfunction through 12 months was 9.4% with SEV and 41.6% with BEV (*p* <0.001 for superiority). In comparison with the BEV group, the SEV group presented at 12 months lower post-TAVR AV mean gradients at 12 months (7.7 vs. 15.7 mmHg, *p* <0.001), larger mean EOA (1.99 vs. 1.50 cm^2^, *p* <0.001), lower rates of HSVD (3.5 vs. 32.8%, *p* <0.001), and lower rates of moderate/severe PPM at 30 days (11.2 vs. 35.3%, *p* <0.001). In a nutshell, the SMART trial showed that patients with severe AS and SAA who underwent TAVR with a SEV-SAV was noninferior to a BEV-IAV with respect to clinical outcomes and were superior with respect to bioprosthetic valve dysfunction and hemodynamic performance through 12 months.

The results of the Small Annuli Randomized to Evolut or Sapien Trial (SMART) trial^15^ confirmed the results of 3 recently published registries (OPERA-TAVI,^3^ TAVI-SMALL 2,^13^ and Bern TAVI^12^), which also showed that, in patients with SAA, implantation of SEV-SAV was associated with a more favorable hemodynamic profile than after BEV-IAV implantation, respectively. It is unclear if this is related only to the supra-annular design of these THVs or if this is also related to the continued expansion of the nitinol frame in SEV-SAVs. If we consider the latter, the fact that the Portico/Navitor THV is mounted on a self-expanding nitinol frame might help with hemodynamics despite its intra-annular design. In our study, we aimed to investigate the comparison between CoreValve/Evolut iterations vs. Portico/Navitor iterations in SAA.

Indeed, differently from the previous studies comparing SEV-SAV with BEV-IAV,^3^^,^^12^^,^^13^^,^^15^ our study comparing CoreValve/Evolut iterations with Portico/Navitor iterations yielded results that reveal that the latter may offer a better hemodynamic profile despite its intra-annular design (as opposed to its BEV counterpart), having a hemodynamic performance equivalent to SEV-SAV. Despite an indexed EOA lower than that of a SEV-SAV in the matched cohort, this difference did not translate into higher mean gradients, lower dimensionless index, or higher rates of HSVD or moderate/severe PPM. While the TAVI-SMALL registry^16^ found SEV-IAV associated with a higher risk for PPM in comparison with SEV-SAV, we did not find this association. Curiously, both our study and the TAVI-SMALL registry^16^ did not find SEV-IAV associated with a higher risk of mortality in the follow-up.

Aligned with our findings, a study by Brown et al.^17^ sought to characterize transvalvular hemodynamics during the first 30 days after TAVR across various THVs while adjusting for annular dimensions and including the Portico valve, SAPIEN 3 Ultra, and Evolut Pro+. A total of 560 patients who underwent TAVR were included, of which 106 (18.9%) received a Portico THV, 176 (31.4%) received a SAPIEN THV, and 278 (49.6%) received an Evolut THV. For Portico THV, the mean gradient on day 0 increased from 6.0 (4.7-9.0) to 7.0 (6.0-10.0) by day 30 (*p* = 0.009). For SAPIEN THV, the mean gradient on day 0 increased from 6.5 (5.0-8.0) to 12.0 (9.0-15.0) by day 30 (*p* <0.001). For Evolut THV, the mean gradient on day 0 increased from 6.0 (5.0-9.0) to 7.2 (5.0-10.0) by day 30 (*p* = 0.001). Adjusting for time and annular diameter in a multivariable mixed effects model, the SAPIEN group had a significantly greater increase in the mean gradients over time than the Evolut reference group (*p* <0.001), while there was no difference in the change of the gradients over time for the Portico group vs. the Evolut group (*p* = 0.874). The authors concluded that, compared with BEVs, SEVs may optimize transvalvular hemodynamics across all annular diameters, independent of their supra-annular and intra-annular designs. The findings by Brown et al.,^17^ along with the results of our present study, might well explain the comparable functional status and quality of life as evidenced by the 1-year KCCQ-12 scores for both groups in our current study. Therefore, we might consider the Portico/Navitor platform as a SEV with “intra-annular design and supra-annular hemodynamics.”

Interestingly, our rates of PPI after TAVR in both unmatched and matched cohorts for both SEV-SAV and SEV-IAV were much lower in comparison with the rates published in other studies in patients with SAA.^3^^,^^11^, ^12^, ^13^^,^^15^ For SEV-SAV, these rates range from 12.1 to 20.6%, while our rates were only 5.5% (unmatched cohort) and 1.2% (matched cohort). The same applies to the SEV-IAV, in which the PPI rates in recent studies^18^, ^19^, ^20^, ^21^ ranged from 15.4 to 27.7%, while our rates were only 8.5% (unmatched cohort) and 8.1% (matched cohort). Our lower PPI rates are highly likely due to the consistent adoption of cusp-overlap view for optimal implantation depth on both TAVR platforms.^22^^,^^23^ The higher rates of PPI in the SEV-IAV arm when compared with the SEV-SAV (matched cohort) might be explained by the initial acquisition of experience (learning curve) with the SEV-SAV platform, and indeed, we observed decreasing rates of PPI over time with this platform in our local experience. Be that as it may, we must strive to reduce the rates of PPI after TAVR with any platform because PPI is associated with a higher risk of all-cause mortality and rehospitalization for heart failure.^24^^,^^25^ The consistent adoption of pre-TAVR measurement of the membranous septum length may be helpful in the optimal implantation of SEV and consequent reduction of PPI rates after TAVR with SEVs.^26^

In our unmatched and matched cohorts, the presence of significant PVL was a rare event (with no cases of severe PVL), and the implementation of external sealing skirts in newer generation devices has evidently reduced the incidence of PVL.^3^

### Study Limitations

Despite the PSM adjustment, the findings of this study may be under the effect of unmeasured confounders due to its observational nature. This work has intrinsic limitations of single-center series, including limited sample size, risk of overinterpretation, and limited external validity. Adjudication of individual events and core imaging laboratory were not components of this study, but the TAVR procedures were carried out at a high-volume, experienced center with standardized procedural and imaging protocols.

A key limitation of this study is the uncertainty of how patients were directed to one therapy or the other. THVs were chosen based on the structural heart team’s preference. For SAA cases, the local team does not choose BEV as an option. When it comes to SEVs for SAA, the members of the team could equally select a SAV or IAV based on the preference of the operators (interventional cardiologist and cardiac surgeon) in each individual case.

Moreover, instead of using the concept of projected/predicted PPM, our study explored NSVD by having PPM measured by Doppler echocardiography. While the former can mitigate the effect of low-flow states on the measurements,^27^ it has the downside of including the extrapolation of the average values of a group (from other studies) to an individual patient (from our own study).^27^^,^^28^

Finally, our findings apply only to the THVs studied and should not be extrapolated to other TAVR platforms. In addition, our results apply only to patients with SAA according to the definition used for inclusion in our study (maximum annulus diameter <23 mm).

## Conclusions

This study provides important insights into the comparative clinical and hemodynamic performance of iterations of the Corevalve/Evolut and Portico/Navitor TAVR platforms in patients with SAA. Both platforms showed equivalent clinical and hemodynamic outcomes characterized by comparable rates of mortality and stroke, comparable transvalvular gradients, and comparable incidences of HSVD and NSVD. Despite these important findings, there is a clear need for further investigation through randomized trials. Therefore, these results should be seen as a springboard for a more comprehensive understanding of TAVR in patients with SAA, guiding future research and patient management strategies in real-world scenarios.

## Ethics Statement

This study was approved by the Institutional Review Board of the University of Pittsburgh (STUDY18120143).

## Funding

The authors have no funding to report.

## Disclosure Statement

I. Sultan receives institutional research support from Abbott, Artivion, Boston Scientific, Edwards, Medtronic, and Terumo Aortic. The other authors had no conflicts to declare.

## Data Availability Statement

The data that support the findings of this study are available from the corresponding author upon reasonable request.
